# Oxidative Stress and AKT-Associated Angiogenesis in a Zebrafish Model and Its Potential Application for Withanolides

**DOI:** 10.3390/cells11060961

**Published:** 2022-03-11

**Authors:** Jen-Yang Tang, Yuan-Bin Cheng, Ya-Ting Chuang, Kun-Han Yang, Fang-Rong Chang, Wangta Liu, Hsueh-Wei Chang

**Affiliations:** 1 School of Post-Baccalaureate Medicine, Kaohsiung Medical University, Kaohsiung 80708, Taiwan; reyata@kmu.edu.tw; 2 Department of Radiation Oncology, Kaohsiung Medical University Hospital, Kaohsiung 80708, Taiwan; 3 Department of Marine Biotechnology and Resources, National Sun Yat-sen University, Kaohsiung 80424, Taiwan; jmb@mail.nsysu.edu.tw; 4 Department of Biomedical Science and Environmental Biology, College of Life Science, Kaohsiung Medical University, Kaohsiung 80708, Taiwan; u107023007@gap.kmu.edu.tw; 5 Graduate Institute of Natural Products, Kaohsiung Medical University, Kaohsiung 80708, Taiwan; kunhan1013@gap.kmu.edu.tw (K.-H.Y.); aaronfrc@kmu.edu.tw (F.-R.C.); 6 Department of Biotechnology, Kaohsiung Medical University, Kaohsiung 80708, Taiwan; 7 Center for Cancer Research, Kaohsiung Medical University, Kaohsiung 80708, Taiwan

**Keywords:** withanolides, oxidative stress, AKT, angiogenesis, zebrafish

## Abstract

Oxidative stress and the AKT serine/threonine kinase (AKT) signaling pathway are essential regulators in cellular migration, metastasis, and angiogenesis. More than 300 withanolides were discovered from the plant family Solanaceae, exhibiting diverse functions. Notably, the relationship between oxidative stress, AKT signaling, and angiogenesis in withanolide treatments lacks comprehensive understanding. Here, we summarize connecting evidence related to oxidative stress, AKT signaling, and angiogenesis in the zebrafish model. A convenient vertebrate model monitored the in vivo effects of developmental and tumor xenograft angiogenesis using zebrafish embryos. The oxidative stress and AKT-signaling-modulating abilities of withanolides were highlighted in cancer treatments, which indicated that further assessments of their angiogenesis-modulating potential are necessary in the future. Moreover, targeting AKT for inhibiting AKT and its AKT signaling shows the potential for anti-migration and anti-angiogenesis purposes for future application to withanolides. This particularly holds for investigating the anti-angiogenetic effects mediated by the oxidative stress and AKT signaling pathways in withanolide-based cancer therapy in the future.

## 1. Introduction

Cell migration is essential in development, angiogenesis, metastasis, and tumorigenesis [1]. Accumulated evidence showed that metastasis shares some signaling pathways with migration and angiogenesis. Several factors affect migration, metastasis, and angiogenesis, such as oxidative stress and the AKT serine/threonine kinase (AKT) signaling pathway. Zebrafish embryos are transparent, and various processes can easily be observed during embryonic development, such as blood vessel formation. Although there are about 300 known withanolides, their angiogenetic effects are scarcely investigated. Moreover, the relationships between oxidative stress, AKT signaling, and angiogenesis in withanolide treatments lack systemic investigation.

This review analyzed the relationship between oxidative stress, AKT, its downstream effects, and angiogenesis from reports utilizing the outcomes of experiments with zebrafish embryos. A particular focus was on developmental angiogenesis and the angiogenesis of xenografted tumor cells within embryos. Oxidative stress, AKT, and its downstream AKT signaling were associated with angiogenesis. Selected withanolides with oxidative stress and AKT-signaling-modulating effects were summarized, but their potential applications to angiogenesis regulation are rarely investigated. Moreover, the contributions of oxidative stress, AKT, and AKT signaling to angiogenesis of withanolides were suggested to be validated in the future. Furthermore, we propose an approach to identify the potential anti-angiogenetic withanolides using inhibitory effects on AKT and AKT signaling as biomarkers and potential drug candidates.

## 2. Oxidative Stress Studies in the Zebrafish Model

Zebrafish exhibit several advantages for the research model. The genes for zebrafish show high similarity to humans, about 70–80% [2]. It is easy to knock interested genes in or out to study human disease [3]. Moreover, zebrafish have similar organs and body parts in humans, and the related genes are highly conserved [4,5]. Zebrafish are smaller than rodents. About every 10 days, zebrafish spawn hundreds of eggs per pairing, while the rodents show a low offspring rate of about 5–10 [6,7,8]. Zebrafish are transparent, and it is easy to observe the embryos’ development process non-invasively; this is not the case for rodent embryos [3]. Finally, zebrafish can tolerate higher concentrations of chemicals or mutagens than rodents [9], making them a valuable tool for drug discovery or environmental toxicology studies. However, the disadvantages of the zebrafish model were previously reported. Some human diseases do not appear in zebrafish. Moreover, some human organs such as the prostate, mammary glands, and lungs do not exist in zebrafish. In these cases, other animal models need to be chosen.

Oxidative-stress-associated studies for several chemical agents and genetic functions are well established in the zebrafish model. Because the zebrafish embryos are transparent, oxidative stress change is easy to observe by direct staining in the following studies. Zebrafish observations validated several pollutant-generating oxidative stress and related cell dysfunctions in vivo. 5-chloro-2-(2,4-dichlorophenoxy (Triclosan) is a broad-spectrum antibacterial agent that is commonly used for cleaning and personal care purposes [10]. The zebrafish model monitors the in vivo effects of triclosan regarding oxidative stress (reactive oxygen species (ROS) generation and mitochondrial membrane depolarization), DNA damage, and caspase-dependent apoptosis [10]. Moreover, this oxidative stress is accompanied by the downregulation of antioxidant signaling enzymes such as manganese superoxide dismutase (MnSOD; SOD2) and glutathione peroxidase (GPX) [10]. Additionally, other antioxidant signaling pathways, such as nuclear factor (erythroid-derived 2)-like 2 (NRF2), kelch-like ECH-associated protein 1 (KEAP1), catalase (CAT), and copper/zinc superoxide dismutase (Cu/ZnSOD; SOD1) in zebrafish [11], are investigated and warrant a detailed examination for oxidative stress experiments.

Similarly, the zebrafish model demonstrates that environmental exposure to polyethylene nanoplastics induced cardiovascular toxicity and oxidative stress [12]. Paraquat, a highly toxic pesticide, induces oxidative stress, apoptosis, and macrophage migration in zebrafish embryos [13]. For normal embryogenesis, the physiological level of oxidative stress controls neural differentiation and maintains normal neuronal development. In contrast, some environmental pollutants, such as benzo[a]pyrene, can increase oxidative stress causing acute neurotoxicity to be examined by the zebrafish model [14]. Therefore, drug-induced oxidative stress can be monitored conveniently in the zebrafish model.

## 3. Angiogenesis Applications in the Zebrafish Model

In addition to migration and angiogenesis in in vitro cell models, a detailed investigation of the in vivo effects is warranted. Zebrafish are applied in several angiogenesis studies, such as anti-angiogenesis drug screening [15,16], angiogenesis disease [17], angiogenesis in regeneration [18], and tumor angiogenesis [19,20]. The molecular mechanisms for vessel development in zebrafish are similar to those in humans [21]. Therefore, the angiogenesis results for humans and zebrafish are closely related. In transparent zebrafish embryos and larvae, vascular development and blood flow are easily visualized [22]. Accordingly, the anti-angiogenesis activity of drug candidates can be monitored using the early developmental stages of zebrafish.

Furthermore, transgenic zebrafish can visualize cells of the vascular system. These can express green fluorescence (enhanced green fluorescent protein; EGFP) in the vascular system; EGFP expression is controlled by the fli1 promoter [23]. Moreover, a high throughput screening method, cognition network technology (CNT), was developed to detect angiogenesis of these fluorescent embryos (*Tg(fli1:EGFP)y1*) for drug screening [16]. Therefore, the zebrafish model is a convenient and reliable tool for studying the development of angiogenesis [24].

## 4. Developmental and Xenograft Tumor Angiogenesis of Zebrafish

**Figure 1:**
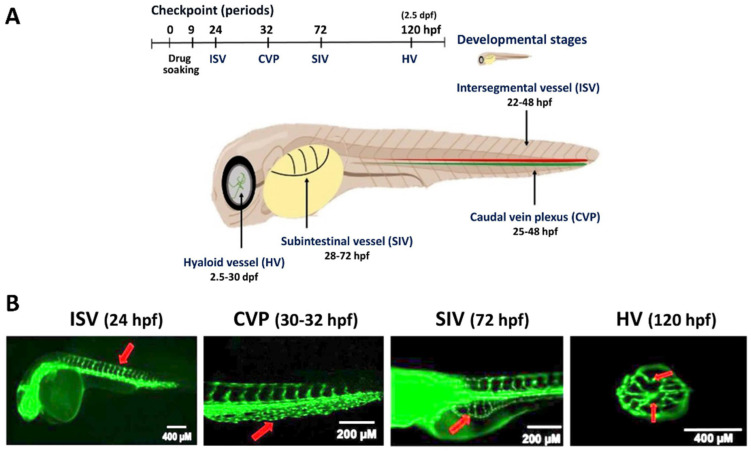
Developmental angiogenesis stages and periods. (**A**) The embryos were subjected to analyses of intersegmental vessel (ISV) formation at 22–48 hpf, caudal vein plexus (CVP) at 25–48 hpf, subintestinal vessel (SIV) at 28–72 hpf, and hyaloid vessel (HV) at 2.5–30 dpf. (**B**) Representative images for different developmental stages. *Tg (fli1: EGFP)* zebrafish embryos were applied for observing the developmental angiogenesis for different stages. The red arrow indicates these structures.

Understanding vascular development is essential for normal embryo development, tumor growth, and metastasis. For normal embryo development, the angiogenesis events for different locations [25] in zebrafish embryos appear at different time windows after fertilization (Figure 1), e.g., intersegmental vessel (ISV) formation at 22–48 h post-fertilization (hpf), caudal vein plexus (CVP) at 25–48 hpf, subintestinal vessel (SIV) at 28–72 hpf, and hyaloid vessel (HV) at 2.5 to 30 days post-fertilization (dpf) [26]. Accordingly, the zebrafish embryo is a convenient model for studying vertebrate angiogenesis in vivo, similar to the human condition.

The primary vascular anatomy is conserved among vertebrates. The distinct locations of blood vessels in zebrafish can be directly compared with their homologs in different vertebrates [27]. Zebrafish inner retinas are nourished with a complex vascular network of HV similar to the human hyaloid and retinal vascular systems [28]. Retina regeneration can also easily be observed in the zebrafish model [29]. Understanding the effects on different stages of developmental angiogenesis is vital for studying human diseases. Irreversible blindness from various eye diseases in humans results from pathological angiogenesis. In children and adults, premature age-related retinopathy impairs vision or causes acquired blindness [30]. Hence, zebrafish represent a convenient animal model for examining developmental angiogenesis and identifying potential therapeutic drug targets.

In addition to developmental angiogenesis, the angiogenesis of xenografted tumor cells provides evidence from different tumor-derived angiogenesis factors. They typically reflect the initiation of tumor angiogenesis and metastasis [31,32]. They are applied to screen the anti-angiogenetic effects of microRNA (miRNAs) [33]. Tumor cells provide a suitable bioassay to screen for anticancer drug compound candidates causing anti-angiogenesis. Therefore, the zebrafish model can examine xenografted tumor angiogenesis and identify potential anticancer drugs.

## 5. Role of Oxidative Stress in the Angiogenesis of the Zebrafish Model

The zebrafish model also validated the oxidative-stress-associated regulation of angiogenesis by several chemicals, toxins, and drugs. Bisphenol A, primarily used in processing polycarbonate plastics, caused developmental vascular toxicity in the zebrafish model, accompanied by dysregulating ISV and SIV formations and common cardinal vein (CCV) remodeling [34]. Acute exposure to saxitoxin, a cyanobacterial toxin, induced oxidative stress such as ROS and triggered DNA damage and apoptosis, reverted by antioxidant *N*-acetylcysteine [35]. Saxitoxin also upregulates vascular-development-related genes such as delta-like canonical notch ligand 4 (DLL4) and vascular endothelial growth factor C (VEGFC) but downregulates VEGFA expression in zebrafish embryos [35]. Nodularin, a cyanobacterial toxin, causes oxidative-stress-mediated developmental toxicity by suppressing embryo hatching and delaying CCV remodeling in zebrafish embryos, accompanied by altering vascular development (DLL4, cadherin 5 (CDH5), VEGFA, and VEGFC) and apoptosis (BAX, BCL2, and caspase 3) gene expressions [36]. Methamphetamine, a central nervous system stimulant, also blocks developmental angiogenesis to generate vessel abnormality in the zebrafish model [37]. Accordingly, the zebrafish model is suitable for validating drug-induced oxidative-stress-associated angiogenesis.

In addition to chemical applications, as mentioned above, genetic investigations of oxidative-stress-associated developmental and tumor xenograft angiogenesis studies were also applied to zebrafish models.

From the viewpoint of developmental angiogenesis, nuclear prelamin A recognition factor-like (narfl) is the upstream regulator for sprouting angiogenesis [38]. Narfl deletion induces oxidative stress and suppresses SIV formation in the zebrafish developmental model [38]. From the viewpoint of tumor xenograft angiogenesis, WNK lysine-deficient protein kinase 1 (WNK1), a regulator for sodium and chloride ion channels, is downstream within the VEGF signaling cascade [39]. Hence, oxidative stress plays an essential role in developmental angiogenesis in zebrafish embryos.

From the viewpoint of tumor xenograft angiogenesis, human hepatoma xenograft angiogenesis upregulates human VEGF and WNK1 and zebrafish wnk1 [40]. In zebrafish experiments, genetic and chemical wnk1 knockdowns suppressed hepatoma tumor xenograft angiogenesis and tumor growth [40]. Additionally, a chemical inhibitor of WNK1 downstream protein, such as oxidative-stress-responsive kinase 1 (OSR1), suppresses tumor-derived vessel formation in zebrafish embryos [40]. Moreover, oxidative stress can induce OSR1 phosphorylation [41]. Therefore, oxidative stress plays a vital role in tumor xenograft angiogenesis in zebrafish embryos.

## 6. AKT Plays a Vital Role in Regulating Migration and Angiogenesis

AKT is a critical downstream regulator of the phosphoinositide-3-kinase/AKT/mechanistic target of the rapamycin kinase (PI3K–AKT–mTOR) pathway, controlling cell proliferation, metabolism, angiogenesis, metastasis, and carcinogenesis [42,43,44,45,46]. AKT is a primary effector of the PI3K/AKT pathway, modulating cancer cell migration [47,48] and angiogenesis [32,49]. Therefore, the potential strategy for modulating AKT in the anti-migration and anti-angiogenesis effects of cancer cells warrants further investigation.

## 7. Role of AKT in Oxidative Stress Studies of the Zebrafish Model

The zebrafish model evaluates the role of AKT in several attempts to study the effects of natural products, toxins, and clinical drugs. In response to high levels of oxidative stress caused by such compounds, PI3K/AKT signaling may be activated [50]. Activated AKT, in turn, also enhanced ROS generation [51]. Quercetin 3-*O*-methyl ether suppressed copper-induced oxidative stress and the cytotoxicity of fetal mice FL83B cells by activating AKT and reducing the survival of copper-treated zebrafish [52]. Coptisine inhibited 2,2′-Azobis(2-amidinopropane) dihydrochloride (AAPH)-induced ROS production, lipid peroxidation, and rapid heart-beating, accompanied by the upregulation of the antioxidant NQO1 and AKT in zebrafish embryos [53]. Schisantherin A provided neuroprotection and ROS-reducing effects against the neurotoxin 6-hydroxydopamine (6-OHDA)-treated zebrafish through upregulating AKT [54]. Pinocembrin-7-methylether inhibited 6-OHDA-induced oxidative stress by activating AKT in neuroblastoma cells. Pinocembrin-7-methylether recovered 6-OHDA-induced locomotor deficiency in the zebrafish model [55]. Therefore, the zebrafish model is a convenient tool to study drug-induced ROS change. Moreover, AKT plays a vital role in regulating ROS and vice versa in zebrafish embryos.

## 8. The AKT Network Plays a Vital Role in Regulating Angiogenesis

AKT exhibits diverse functions in regulating many AKT-responsive proteins that form the AKT signaling network in cooperatively regulating angiogenesis. Here, we focused on three parts, including the network between AKT, matrix metalloproteinases-2 and 9 (MMP-2/9), epithelial–mesenchymal transition (EMT), and migration (Section 8.1), the regulation of AKT to several migration- and angiogenesis-related genes to modulate EMT (Section 8.2), and the AKT network, Warburg effect, migration, and angiogenesis (Section 8.3). These are detailed in Table 1.

### 8.1. The Network between AKT, MMP-2/9, EMT, and Migration

The network between AKT and MMP-2/9 and between AKT and EMT was elucidated by several drug treatment studies as follows.

MMPs, a kind of extracellular matrix, are involved in tumorigenesis and induce angiogenesis, resulting in the invasion and metastasis of tumor cells [81] (Table 1). Using inhibitors of MMP-2 (ARP100) and MMP-9 (AG-L-66085), the migration in metastatic Y79 retinoblastoma cells was inhibited [56], demonstrating that MMP-2 and MMP-9 could promote Y79 cancer cell migration. Moreover, AKT may either activate MMP-2 or MMP-9 in different treatments. *Salvia miltiorrhiza*-derived tanshinone IIA exhibits an antimigration effect by suppressing MMP-9 and AKT signaling in the tumor necrosis factor α-induced migration of human aortic smooth muscle cells [57]. siRNA knockdown of eukaryotic translation elongation factor 1 α2 (eEF1A2), a protein translation factor overexpressed in cancer cells, suppresses the migration and invasion of pancreatic cancer cells by downregulating MMP-9 and inactivating AKT [47]. This result indicates that the eEF1A2 protein with an MMP-9 and AKT activating ability can improve the migration of pancreatic cancer cells. Hence, AKT and MMP-2/9 cooperatively regulate cell migration (Table 1).

Moreover, AKT also upregulates EMT signaling [82,83], which controls the migration and invasion of cancer cells. Several drugs inhibit EMT and cancer cell migration (Table 1). Through EMT inhibition, paeonol [58] and emodin [59] inhibit the migration and invasion of pancreas and colon cancer cells. Therefore, AKT exhibits the network connection between MMP-2/9, EMT, and migration (Table 1).

### 8.2. AKT Regulates Several Migration- and Angiogenesis-Related Genes to Modulate EMT

Several migration- and angiogenesis-related genes, such as mitogen-activated protein kinase (MAPK), platelet-derived growth factor (PDGF), VEGF, nitric oxide synthase (NOS), epidermal growth factor receptor (EGFR), and heat shock protein 90 (HSP90), are reported. Their migration and angiogenesis signaling proteins may be regulated by AKT. These AKT regulations to migration and angiogenesis genes were elucidated by several studies as follows.

The interplay between MAPK and AKT is reported in several studies [60,61] (Table 1). MAPK also tightly regulates MMP-2/9 activity. For instance, crude pomegranate POMx extract inhibits the migration and invasion of oral cancer cells by suppressing MMP-2/9 activity and EMT expression in an ERK1/2-dependent manner [62]. POMx-induced antimigration activity, such as Transwell migration and MMP-2/9 activity, is suppressed using an ERK inhibitor. PDGF activates AKT signaling to promote the proliferation of arterial smooth muscle cells [63]. High glucose upregulates VEGF-C expression via AKT and ROS in prostate cancer cells [64]. AKT activates endothelial NOS (eNOS) to generate nitric oxide (NO) in endothelial cells [65]. EGF induces AKT phosphorylation in promoting the migration of oral cancer SAS cells, reverted by AKT inhibitor MK2206 [66]. EGFR induces EMT in breast cancer cells [67]. AKT improves FOXO1 phosphorylation in prostate cancer cells [68]. Accordingly, drug-induced AKT activation can regulate migration and angiogenesis signaling (Table 1).

Moreover, HSP90 is an activator of AKT (Table 1). Circular RNA (circSTK40) overexpression upregulates HSP90 to activate AKT for improving HSP90–AKT interactions in breast cancer cells [69]. HSP90 promotes EMT, invasion, and migration in colorectal cancer, reverted by the chemical (ganetespib) and genetic knockdown of HSP90 [70]. These findings suggest that HSP90 may activate AKT to promote EMT signaling and migration.

**Figure 2:**
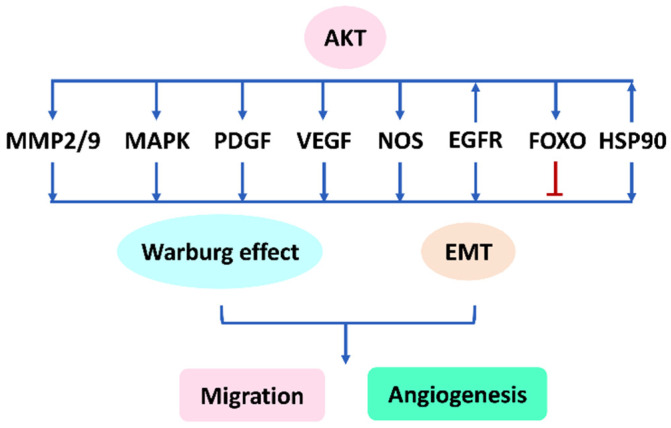
AKT signaling network affects migration and angiogenesis. This flow chart summarizes Section 1, Section 2, Section 3, Section 4, Section 5, Section 6, Section 7 and Section 8.

Therefore, AKT may regulate AKT signaling, including MAPK, PDGF, VEGF, NOS, EGFR, FOXO, and HSP90, to modulate EMT (Figure 2, Table 1).

### 8.3. AKT Network, Warburg Effect, Migration, and Angiogenesis

Warburg effect is a typical cancer character that cancer cell proliferation prefers to anaerobically metabolize glucose in the absence and presence of oxygen [84]. The networks between the Warburg effect, migration, and angiogenesis, between AKT and the Warburg effect, and between AKT signaling and the Warburg effect was elucidated by several studies as follows.

The Warburg effect can enhance migration and angiogenesis (Table 1). Pyruvate dehydrogenase kinase 1 (PDK1) induces the Warburg effect to improve the migration of lung cancer cells [71]. The Warburg effect benefits angiogenesis because endothelial cells provide high glycolytic activity [72]. Moreover, the Warburg effect may generate a large amount of lactate and uptake by tumor endothelial cells to induce angiogenesis via monocarboxylate transporter 1 (MCT1) [73]. In contrast, knockdown MCT1 inhibits tumor angiogenesis. Accordingly, modulation of the Warburg effect may inhibit migration and angiogenesis (Table 1).

AKT induces the Warburg effect in cancer cells [74]. Furthermore, AKT network signaling proteins (Section 8.1 and Section 8.2) may regulate the Warburg effect (Table 1). p38γ MAPK is necessary for the Warburg effect in pancreatic carcinogenesis [75]. PDGF activates PI3K/AKT signaling to induce the Warburg effect in arterial smooth muscle cells, reverted by PI3K inhibitor [63]. Moreover, fructose 1,6 bisphosphate (F1,6BP), a glycolysis enzyme, can activate EGFR to promote the Warburg effect (lactate secretion) in triple-negative breast cancer cells [76]. NO generated by NOS can improve the Warburg effect by upregulating hexokinase 2 (HK2) expression in ovarian cancer cells [77]. FOXO3a suppresses the Warburg effect in glioblastoma cells, which is reverted by FOXO3a silencing [78]. Hypoxia-inducible factor 1α (HIF-1α) induces hypoxia response and VEGF expression in lung cancer cells, where VEGF regulates the Warburg effect [79]. In liver cancer cells, HSP90 can activate pyruvate kinase muscle isozyme 2 (PKM2) [80], which is an activator for Warburg metabolism in cancer cells [85]. These studies support the rationale that AKT signaling contributes to modulating the Warburg effect.

Therefore, AKT may regulate its network signaling to modulate migration and angiogenesis (Figure 2, Table 1).

## 9. Overview of the Current Developments for Oxidative Stress, AKT, AKT Signaling, and Angiogenesis of Selected Withanolides

**Figure 3:**
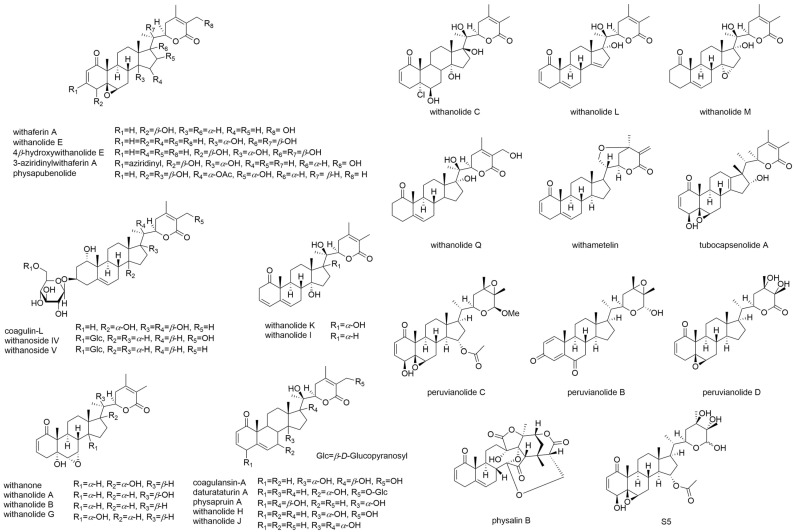
Structures for the selected withanolides mentioned in this review.

Solanaceae plants have an abundance of withanolides, steroid-containing C_28_ compounds with a δ-lactone group located at the C-20 site of the side chain [86]. More than 300 withanolides are found from natural sources [87]. In the present review, we focus on bioactive compounds (Figure 3), such as withaferin A, withanone, withanolide–WS-1 (aliphatic ketone), withanolide–WS-2 (aliphatic ester), withanosides IV and V [88,89,90], withanolides A-C [91,92], E-M, and Q, together with 4β-hydroxywithanolide E [93]. Other withanolides (Figure 3) with antioxidant or pro-oxidant effects (Table 2), AKT inactivation and activation effects (Table 3), and AKT-signaling-pathway-regulating effects (Table 4), were also included and are described below.

In general, compounds with phenolic moieties, such as flavonoids and lignans, might possess free radicals [94]. Free radicals on oxygen atoms can resonate with benzene rings, stabilizing them and exerting antioxidant effects. Withanolides (Figure 3) do not possess a phenolic moiety; thus, the antioxidant potential of some of these types of compounds remains unclear.

## 10. Withanolides May Have Dual Functions in Regulating Oxidative Stress

Several kinds of crude extracts from Solanaceae plants are rich in antioxidants. *Withania somnifera* extracts exhibited a free radical scavenging ability [95]. Aqueous extracts of *W. somnifera* roots possess antioxidant properties which protect the hydrogen-peroxide-induced antiproliferation of adrenal pheochromocytoma cells (PC12) [96]. Moreover, several withanolides can be isolated from Solanaceae plant extracts and exhibit antioxidant abilities based on biochemical assays. Withaferin A and withanolide A exhibit antioxidant properties [95,97]. Withanone [98], withanolide B, and withanoside V exhibit a di(phenyl)-(2,4,6-trinitrophenyl)iminoazanium (DPPH) antioxidant ability [97]. Accordingly, some kinds of Solanaceae plant extracts and withanolides have antioxidant potential.

Notably, the antioxidants provide bifunctional effects on oxidative stress homeostasis. Antioxidants at physiological concentrations show a ROS-reducing impact, but they show ROS-inducing effects at higher lethal concentrations [99]. Hence, the possible bifunctional effects, i.e., antioxidant or pro-oxidant, of withanolides with antioxidants were discussed as follows (Table 2).

Although the DPPH antioxidant ability of several withanolides is rarely investigated in biochemical assays, different withanolides may exhibit antioxidant or pro-oxidant properties in the cell model (Table 2). Coagulin-L, physalin B, and coagulansin-A studies reported antioxidant effects. Coagulin-L exhibited antioxidant and immunosuppression to Toll-like receptor 4-induced DNA, protein, and lipid peroxidation by suppressing ROS generation within mitochondria in macrophages [100]. Physalin B can activate antioxidant signaling related to NRF2 target gene expression to suppress hepatic-injury-induced oxidative stress [101]. Coagulansin-A inhibited oxidative stress, inflammation, and DNA damage in embryonic development [102]. Withanone can suppress *N*-methyl-d-aspartate (NMDA)-induced ROS, apoptosis, and DNA damage in neuron-like cells [98]. Accordingly, some withanolides exert antioxidant effects on normal and cancer cell treatments.

In contrast, several withanolides exhibit pro-oxidant properties and induce oxidative stress with anticancer effects (Table 2). Tubocapsenolide A induces ROS and suppresses glutathione generation to trigger apoptosis in breast cancer cells [103]. Physapubenolide suppresses mitochondrial membrane potentials and induces ROS, leading to apoptosis and autophagy in breast cancer cells [104]. Withanolide C [91] and physapruin A [105] induces antiproliferation and ROS generation in breast cancer cells. 4β-Hydroxywithanolide E shows selective antiproliferation and promotes oxidative stress and DNA damage to oral cancer cells [106]. Hence, some withanolides have pro-oxidant effects on cancer cell treatments.

In contrast, some withanolides, such as physalin A and withaferin A, have dual functions in regulating oxidative stress (Table 2), i.e., they exhibited both antioxidant and pro-oxidant properties in different studies. Physalin A may exhibit cellular antioxidant effects. Physalin A suppresses lipopolysaccharide-induced inflammation in murine macrophage RAW 264.7 cells by upregulating antioxidant signaling gene expressions such as superoxide dismutase, catalase, and glutathione peroxidase 1 [107]. In contrast, physalin A may exert cellular pro-oxidant effects. Physalin A induces antiproliferation and ROS generation in melanoma [108] and lung [109] cancer cells. Similarly, withanolide A shows an antioxidant effect by inducing glutathione, a cellular antioxidant, in neuronal cells to prevent neurodegeneration [110]. In contrast, withaferin A shows a pro-oxidant effect by generating ROS and apoptosis in leukemia [111], oral [112], and bladder [113] cancer cells, reverted by the antioxidant *N*-acetylcysteine. Accordingly, some withanolides have both antioxidant and pro-oxidant effects on different cell treatments.

## 11. AKT-Modulating Effects of Withanolides

Several withanolide studies reported an antiproliferation- and apoptosis-inducible function against cancer cells by regulating the AKT signaling pathway (Table 3). Tubocapsenolide A inhibits proliferation and induces G1 arrest and apoptosis in breast cancer cells by inactivating HSP90–HSP70 and AKT [103]. Withaferin A induces apoptosis and antiproliferation in head and neck [114], ovarian [115], lung [116], and melanoma [117] cancer cells, accompanied by inactivating AKT. Accordingly, some withanolides can inactivate AKT to induce the apoptosis of cancer cells.

Furthermore, withanolide metabolites (Table 3) such as withametelin and coagulansin-A, isolated from *Datura innoxia* and *Withania coagulans*, induce antiproliferation and apoptosis of acute myeloid leukemia (AML) cells by inactivating PI3K/AKT [118]. Daturataturin A, a withanolide in *Datura metel* L., induces autophagy and senescence but inhibits interleukin-17-induced migration in keratinocytes HaCaT by inhibiting PI3K–AKT–mTOR signaling [119]. Furthermore, physapubenolide suppresses the Warburg effect and induces apoptosis in liver cancer cells by inhibiting AKT, which is reverted by AKT overexpression [120]. Hence, some withanolide metabolites also can inactivate AKT to induce apoptosis and non-apoptosis effects on cancer cells (Table 3).

**Table 3:** Withanolides exhibit AKT inactivation and activation of cells.

Drugs	AKT	Cells	References
Withaferin A	inactivation	head and neck cancer	[114]
		ovarian cancer	[115]
		lung cancer	[116]
		melanoma	[117]
	inactivation/activation	breast cancer	[121]
Tubocapsenolide A	inactivation	breast cancer	[103]
Withametelin	inactivation	myeloid leukemia	[118]
Coagulansin-A	inactivation	myeloid leukemia	[118]
Daturataturin A	inactivation	keratinocytes	[119]
Physapubenolide	inactivation	liver cancer	[120]

Some withanolides may exhibit dual functions, inactivating and activating AKT (Table 3). Withaferin A produces differential dose-dependent responses regarding AKT in breast cancer cells, i.e., low and high doses of withaferin A activated and inactivated AKT, respectively [121]. Withaferin A inhibits simulated ischemia/reperfusion-induced ROS, reverted by an AKT inhibitor [122]. Therefore, different withanolides may exert differential AKT activation or inactivation effects for different cellular responses (Table 3). However, the angiogenesis effects of withanolides are rarely investigated, and warrant detailed validation in the future.

## 12. The Role of Oxidative Stress and AKT in Regulating Angiogenesis by Withanolides Needs Further Investigation

As described above, withanolides may have ROS- and AKT-modulating effects (Section 10 and Section 11), contributing to angiogenesis (Section 5 and Section 8). Withaferin A, a potent angiogenesis inhibitor [123], can target EMT proteins such as vimentin, showing an anti-angiogenesis effect for a corneal neovascularization test [124]. Withanolide D inhibits angiogenic sprouting in endothelial cells [125]. However, the role of oxidative stress and AKT studies of withanolides rarely investigate its potential effects on angiogenesis.

## 13. AKT Inhibitors Have the Potential for Anti-Migration and Anti-Angiogenesis Effects, but These Effects Are Rarely Reported in Withanolides

The AKT inhibitors derived from withanolides are rarely investigated. To elucidate the anti-migration and anti-angiogenesis functions for AKT inhibitors, we first used several non-withanolides as examples. AKT is a modulator of migration and angiogenesis. Therefore, AKT inhibition may downregulate cancer cell migration and angiogenesis [126,127]. Kaempferol suppresses renal cancer cell migration/invasion by AKT inhibition [128]. Cantharidin inhibits AKT signaling to downregulate gastric cancer cell migration/invasion [129].

Similarly, several studies report that AKT is associated with cancer cell migration and is downregulated by AKT inhibitors. A conditioned medium from cancer-associated fibroblasts stimulates the scattering and invasion of cancer cells and induces AKT phosphorylation previously suppressed by AKT inhibitors [130]. Nerve growth factors induce AKT phosphorylation in oral cancer cells to improve cell migration in an AKT-dependent manner blocked by an AKT inhibitor (MK2206) [131]. Selenadiazole derivatives show anti-angiogenesis effects to breast-cancer-xenografted mice by downregulating AKT signaling [132]. AKT promotes pathological angiogenesis in a retinopathy model [133]. In contrast, retinal neovascularization is suppressed by 1L-6-hydroxymethyl-chiro-inositol2-(R)-2-*O*-methyl-3-Ooctadecylcarbonate, an AKT inhibitor [133]. Accordingly, these AKT-inhibiting drugs exhibit inhibitory effects on cancer cell migration and angiogenesis.

Notably, some withanolides are potential AKT inhibitors, but their impacts on angiogenesis remain unclear. Withaferin A was reported to inhibit AKT and EMT signaling [134], although angiogenesis effects were not investigated. However, most angiogenesis studies still rarely report the AKT-modulating effects of withanolides. Detailed investigations exploring the role of AKT in regulating angiogenesis by withanolides are warranted in the future. Moreover, withanolides with AKT inhibition abilities are highly suggested for anti-angiogenesis validation, as is angiogenesis with AKT activating ability. Alternatively, withanolides with AKT activating ability may exhibit the opposite effect to angiogenesis, although this warrants detailed investigation.

Therefore, as described (Section 11), some withanolides showing AKT inhibitory effects have the potential for anti-migration and anti-angiogenesis but need to be further examined.

## 14. AKT Signaling Inhibitors Have the Potential for Anti-Migration and Anti-Angiogenesis Effects, but These Effects Are Rarely Reported in Withanolides

As described above, AKT can control migration and angiogenesis; therefore, the AKT responsive downstream (AKT signaling) has the potential to show similar effects. Hence, AKT signaling may play vital roles in regulating migration and angiogenesis, such as MAPK, PDGF, VEGF, NOS, EGFR, FOXO, and HSP90 (Figure 2). Inhibitors for these AKT signaling pathways may also exhibit anti-migration and anti-angiogenesis effects; however, the modulating effects to AKT signaling by withanolides are still rarely investigated.

Notably, several withanolides demonstrate connections to AKT signaling (MAPK, PDGF, VEGF, NOS, EGFR, FOXO, and HSP90), although angiogenetic effects are rarely investigated (Table 4). Withanolides such as withametelin and coagulansin-A downregulate MAPK (ERK1/2) in acute myeloid leukemia cells, causing antiproliferation and apoptosis [118]. Withaferin A exhibitsd antifibrogenic effects by suppressing PDGF-induced liver fibrosis in JS1 cells [135]. Withaferin A downregulates VEGF in Ehrlich ascites tumor cells [136]. Peruvianolide B, peruvianolide C, and peruvianolide D show inhibitory effects by NO, with supporting evidence of decreasing NO release and fitting molecular docking to inducible NOS (iNOS) [137]. S5, the inseparable epimeric withanolide from *Physalis pubescens* L., shows the antiproliferation of melanoma cells by upregulating EGFR [138]. Withaferin A activates FOXO3a-induced cell death for prostate cancer cells, where FOXO3a may suppress the Warburg effect [79] to inhibit angiogenesis. Accordingly, some withanolides may modulate AKT signaling; therefore, their regulating potentials for angiogenesis warrant detailed investigation in the future.

Based on the structure–activity relationship, withaferin A, 3-aziridinylwithaferin A, withanolide E, and 4-hydroxywithanolide E are directly bound to HSP90 and inhibit HSP90 activity from inactivating AKT in pancreatic cancer cells [139]. Several withanolide-induced breast [140] and pancreatic [139] cancer cell deaths are correlated with their ability to inhibit HSP90. A molecular docking study for several withanolide analogs identified HSP90 as their potential target [141]. HSP90 improved angiogenesis and tumor growth [142]. In contrast, *HSP90* inhibitors inactivated *AKT*, showing anti-angiogenetic effects [143]. However, the modulating effects of angiogenesis on AKT signaling, such as HSP90 with withanolides, are still rarely investigated. Therefore, withanolides with an AKT signaling inhibition ability are highly recommended for further anti-angiogenesis validation (Table 4).

## 15. Conclusions

Oxidative stress and the AKT signaling pathway are well-known modulators for angiogenesis. Although more than 300 withanolides were discovered in Solanaceae plants, their angiogenesis, oxidative stress effects, and contributions to AKT and AKT signaling in anticancer and toxicologic effects are rarely investigated.

As mentioned above, the oxidative stress, AKT, and AKT signaling impacts are well organized and connected to angiogenesis in disease and tumor developments. Withanolides exhibit diverse pro-oxidant and antioxidant effects, showing differential regulation to protect or generate oxidative stress. Accordingly, detailed investigations of this oxidative-stress-modulating function of withanolides to regulate angiogenesis are warranted in the future.

**Figure 4:**
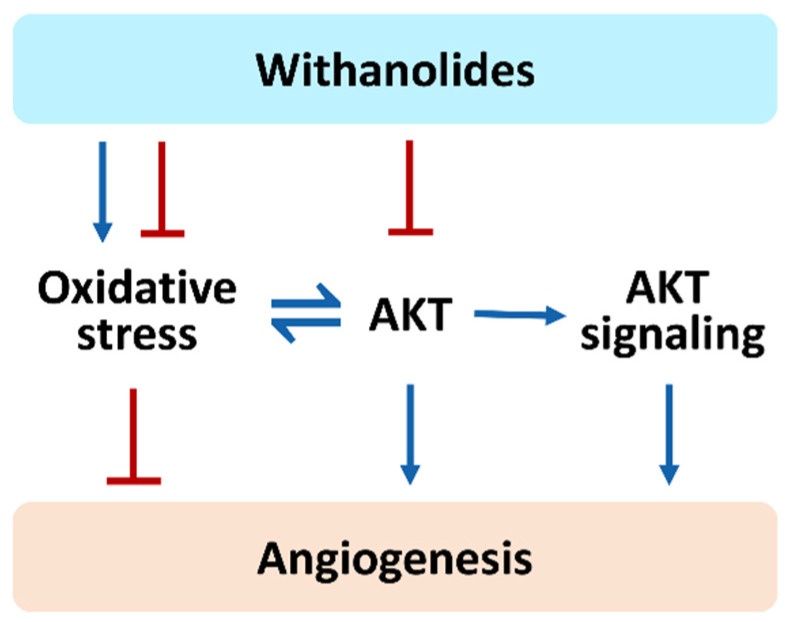
Hypothesis: Withanolides affecting oxidative stress and AKT signaling may modulate angiogenesis, although this is rarely reported. Detailed investigation is warranted to examine the potential angiogenesis-modulating effects of withanolides in the future.

In addition to the AKT regulating ability, we also summarized that withanolides exhibit modulating effects on AKT signaling, including MAPK, PDGF, VEGF, NOS, EGFR, FOXO, and HSP90, but their angiogenesis effects are rarely investigated. Accordingly, withanolides with a modulating ability for AKT signaling are expected to show potential regulation to angiogenesis. Hence, we proposed a hypothesis that the functions of oxidative stress, AKT, and AKT signaling in withanolide studies may also provide potential anti-cancer applications of regulating angiogenetic effects (Figure 4) through AKT and AKT signaling inhibition.

The zebrafish model benefits the study of developmental and tumor xenograft angiogenesis, as well as xenograft tumor formation associating several diseases and cancer development. The contribution of this review is to propose a novel idea to apply the zebrafish model to investigate the angiogenesis-regulating ability of withanolides and explore the in vivo roles of oxidative stress, AKT, and AKT signaling. This strategy could make anti-angiogenetic functions of withanolides useful in cancer and disease treatments using the zebrafish model.

## Figures and Tables

**Table 1:** Summary for the AKT-regulating–AKT-signaling network and modulating migration and angiogenesis.

Drugs/Proteins	Activation	Inactivation	Migration Effects	Cells	References
Section 8.1. The Network between AKT, MMP-2/9, EMT, and Migration					
ARP100, AG-L-66085		MMP-2/9	inhibit	retinoblastoma	[56]
Tanshinone IIA		AKT, MMP-9	inhibit	aortic smooth muscle	[57]
siRNA eEF1A2		AKT, MMP-9	inhibit	pancreatic cancer	[47]
Paeonol		EMT	inhibit	pancreas cancer	[58]
Emodin		EMT	inhibit	colon cancer	[59]
Section 8.2. AKT Regulates Several Migration- and Angiogenesis-Related Genes to Modulate EMT					
MAPK	AKT			fibroblast, cancer	[60,61]
POMx		MMP-2/9, EMT	inhibit	oral cancer	[62]
PDGF	AKT		promote	aortic smooth muscle	[63]
High glucose	AKT, VEGF-C			prostate cancer	[64]
AKT	eNOS			endothelial cells	[65]
EGF	AKT		promote	oral cancer	[66]
EGFR	EMT			breast cancer	[67]
AKT	FOXO1			prostate cancer	[68]
circSTK40	HSP90, AKT			breast cancer	[69]
HSP90	EMT		promote	colorectal cancer	[70]
Section 8.3. AKT Network, Warburg Effect, Migration, and Angiogenesis					
PDK1	Warburg effect		promote	lung cancer	[71]
High glycolysis	Warburg effect		angiogenesis	endothelial	[72]
MCT1	Warburg effect		angiogenesis	endothelial	[73]
AKT	Warburg effect			cancer	[74]
p38γ MAPK	Warburg effect			pancreatic cancer	[75]
PDGF, AKT	Warburg effect			aortic smooth muscle	[63]
F1,6BP, EGFR	Warburg effect			breast cancer	[76]
NOS, HK2	Warburg effect			ovarian cancer	[77]
FOXO3a		Warburg effect		glioblastoma	[78]
HIF-1α, VEGF	Warburg effect			lung cancer	[79]
HSP90, PKM2	Warburg effect			liver cancer	[80]

**Table 2:** Withanolides exhibit antioxidant and/or pro-oxidant effects.

Drugs	ROS	References
Coagulin-L	decrease	[100]
Physalin B	decrease	[101]
Coagulansin-A	decrease	[102]
Withanone	decrease	[98]
Tubocapsenolide A	increase	[103]
Physapubenolide	increase	[104]
Withanolide C	increase	[91]
Physapruin A	increase	[105]
4β-Hydroxywithanolide E	increase	[106]
Physalin A	decrease/increase	[107,108,109]
Withaferin A	decrease/increase	[110,111,112,113]

**Table 4:** Withanolides that can regulate the AKT signaling pathway.

Drugs	AKT Signaling	References
Withametelin	MAPK (ERK1/2)	[118]
Coagulansin-A		
Withaferin A	PDGF	[135]
	VEGF	[136]
	FOXO3a	[79]
	HSP90	[139]
Peruvianolide B	NOS	[137]
Peruvianolide C		
Peruvianolide D		
S5	EGFR	[138]
3-Aziridinylwithaferin A	HSP90	[139]
Withanolide E		
4-Hydroxywithanolide E		

## Data Availability

Not applicable.
